# Decoding the Rarity: A Unique Case of Clear-Cell Variant of Oral Squamous Cell Carcinoma

**DOI:** 10.7759/cureus.68389

**Published:** 2024-09-01

**Authors:** Anwesha Paul, Arunit Chatterjee, Rudra Prasad Chatterjee, Sudeshna Bagchi, Mehebuba Sultana, Sangeeta Sinha, Mousumi Pal, Sanjeet Das

**Affiliations:** 1 Oral and Maxillofacial Pathology, Guru Nanak Institute of Dental Sciences & Research, Kolkata, IND

**Keywords:** cd10 marker, s100, p63, pan-ck, immunohistochemistry(ihc), mucicarmine, periodic acid-schiff (pas), clear cell tumors, oral squamous cell carcinoma, oral malignancy

## Abstract

The clear-cell variant of oral squamous cell carcinoma is an extremely rare histological variant and an incompletely understood entity. Clear cell appearance in squamous cell carcinoma may be attributed to hydropic degeneration of neoplastic cells. We report a case of a 32-year-old male patient who presented with an ulceroproliferative growth in the left maxillary posterior region on the hard palate and gingiva, obliterating the buccal vestibule. Histopathologic examination revealed thick anastomosing strands of round to ovoid neoplastic cells with predominantly clear cytoplasm and marked cellular and nuclear pleomorphism infiltrating into the fibro-cellular connective tissue stroma. Special staining and immunohistochemistry (IHC) were performed to rule out the differentials of clear-cell variants of different sites such as salivary gland, odontogenic origin, and metastatic tumors. The clear cells were negative for periodic acid Schiff (PAS) and mucicarmine. The malignant clear cells showed positive reactions with IHC markers pan-cytokeratin and P63 and yielded negative results for S100 and CD10, confirming the diagnosis as a clear-cell variant of oral squamous cell carcinoma. We emphasize the importance of prompt and comprehensive diagnostic work-up to identify this rare, aggressive, and possibly fatal neoplasm.

## Introduction

Oral squamous cell carcinoma (OSCC) is a major global health burden accounting for almost 90% of all head and neck malignancies [1]. According to GLOBOCAN (Global Cancer Observatory) 2022 estimates produced by the International Agency for Research on Cancer (IARC), squamous cell carcinoma (SCC) in the lip and oral cavity alone accounted for 389846 cases and 188438 deaths [2]. OSCC is divided into well, moderate, or poorly differentiated based on histologic grade. Occasionally, histopathological variants aggregating to 10-15% of cases are noted and these include verrucous, papillary, spindle cell (sarcomatoid), basaloid, and adenosquamous carcinoma [3].

Clear-cell variant of SCC is an exceedingly rare entity, first reported by Kuo in 1980 as a cutaneous tumor [4]. Its presence in the oral cavity is extremely uncommon with the first case reported in 2012 by Frazier et al. [5] and only 19 cases reported so far in the English literature [5-21]. Clear cell appearance in SCC may be attributed to hydropic degeneration of neoplastic cells due to the accumulation of intracellular fluids and not glycogen, mucin, or lipid [14]. It is also suggested that clear cell appearance may reflect progressive degenerative changes indicating aggressive behavior, early metastasis, and poor prognosis of advanced cases of OSCC [17].

This report describes a rare case of a clear-cell variant of OSCC in the maxillary gingiva and hard palate of a young male patient.

## Case presentation

A 32-year-old male patient presented with the chief complaint of bleeding from his mouth for the last two weeks. He stated that the preliminary symptom was toothache in the left maxillary posterior region two months prior to presentation, following which a swelling developed in the region. He had been prescribed antibiotics and analgesics but they provided no respite from the symptoms. Over the course of the two months, he noted a progressive decrease in mouth opening and occasional bleeding, especially during food intake. The patient had a habit of consuming tobacco in smoke and smokeless forms multiple times a day for the last 10 years.

**Figure 1:**
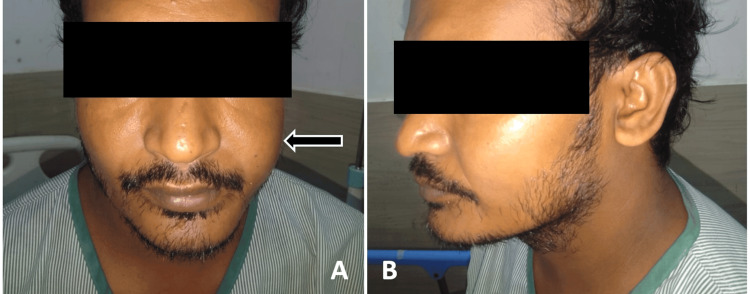
Extraoral photograph from front (A) and left side profile (B) showing mild facial asymmetry and diffuse swelling in the left middle third of face (arrow)

On extraoral examination, mild facial asymmetry was noted due to diffuse swelling in the left middle third of the face (Figure 1). Epiphora was present. No sign of paraesthesia was detected. Ipsilateral level IB and II lymph nodes were palpable, measuring less than 1 cm, firm, non-tender, and not fixed to underlying structures and overlying skin.

**Figure 2:**
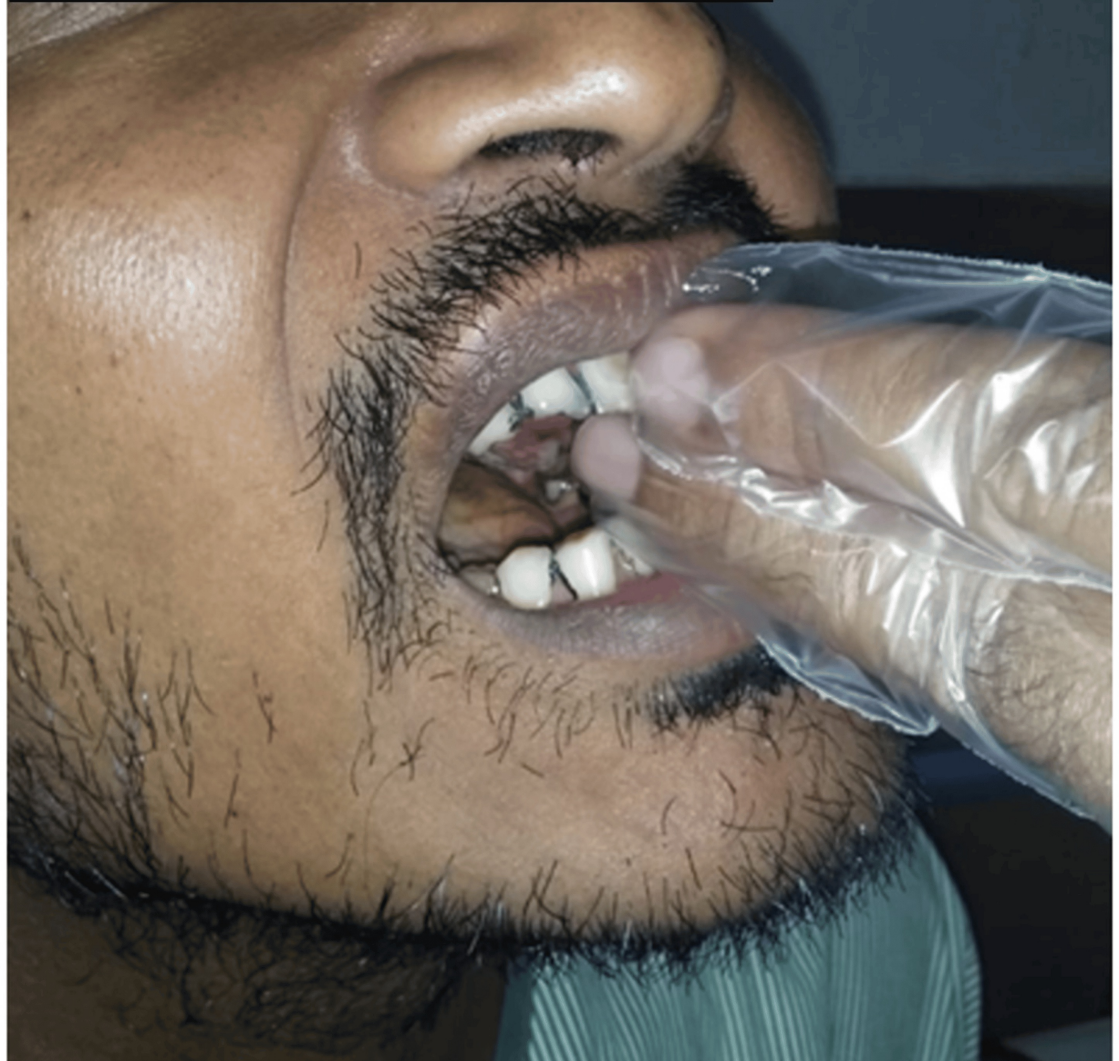
Presence of trismus with one finger mouth opening

**Figure 3:**
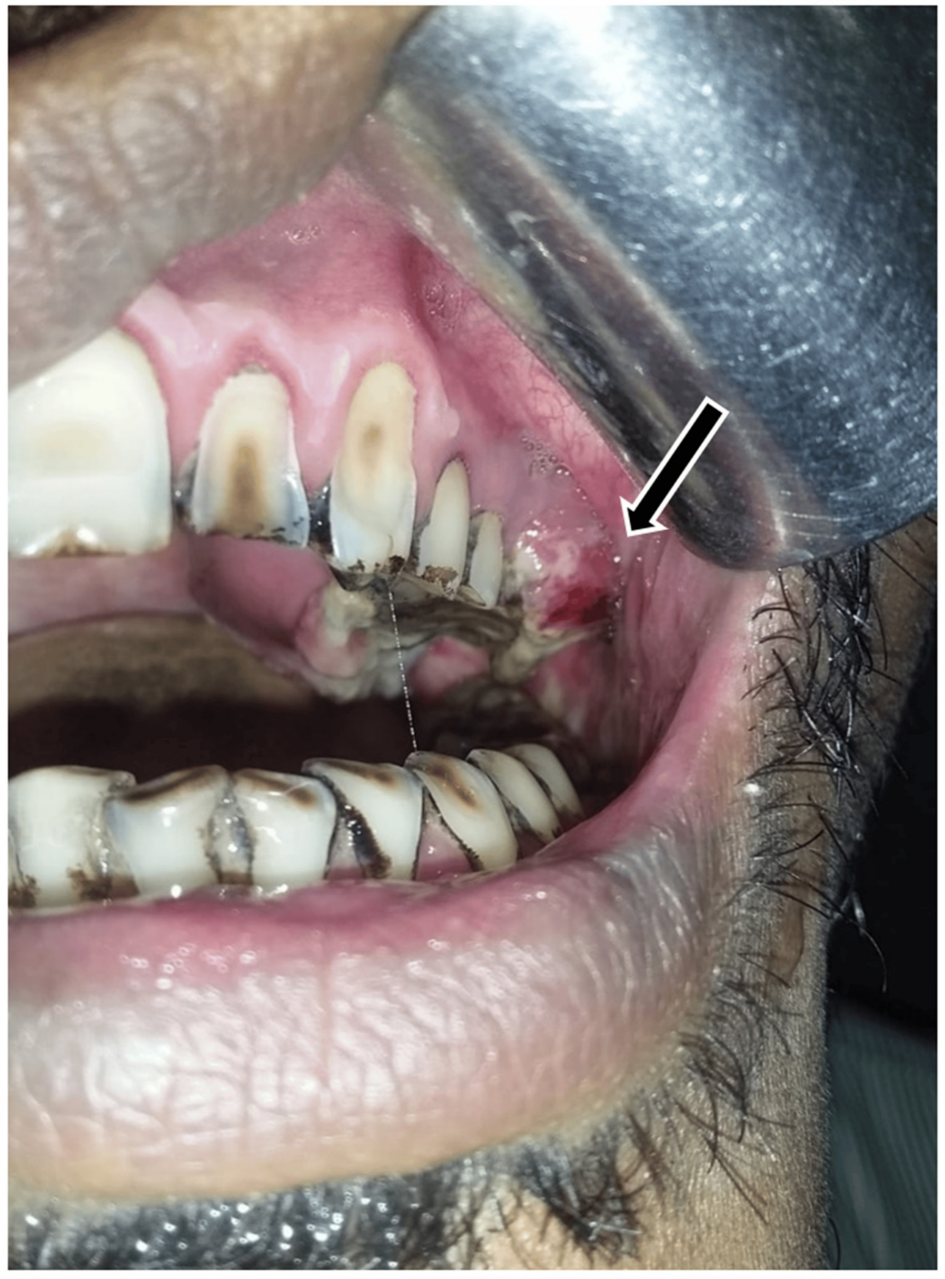
Intraoral photograph showing an exophytic, ulceroproliferative growth appearing greyish-white in colour with interspersed erythematous areas and covered by a fibrinous slough in the left maxillary posterior region on the hard palate and gingiva, obliterating the buccal vestibule distal to 25 (arrow)

Presence of trismus was noted, with one finger mouth opening measuring approximately 15 mm interincisal distance (Figure 2). On intraoral inspection, an exophytic, ulceroproliferative growth with a rough granular surface, measuring about 3 cm x 5 cm was observed in the left maxillary posterior region on the hard palate and gingiva, obliterating the buccal vestibule distal to 25. The lesion appeared greyish-white in color with interspersed erythematous areas and covered by a fibrinous slough (Figure 3). Spontaneous hemorrhage was also present. On palpation, the lesion was noted to be soft to firm, tender, and with indurated margins.

**Figure 4:**
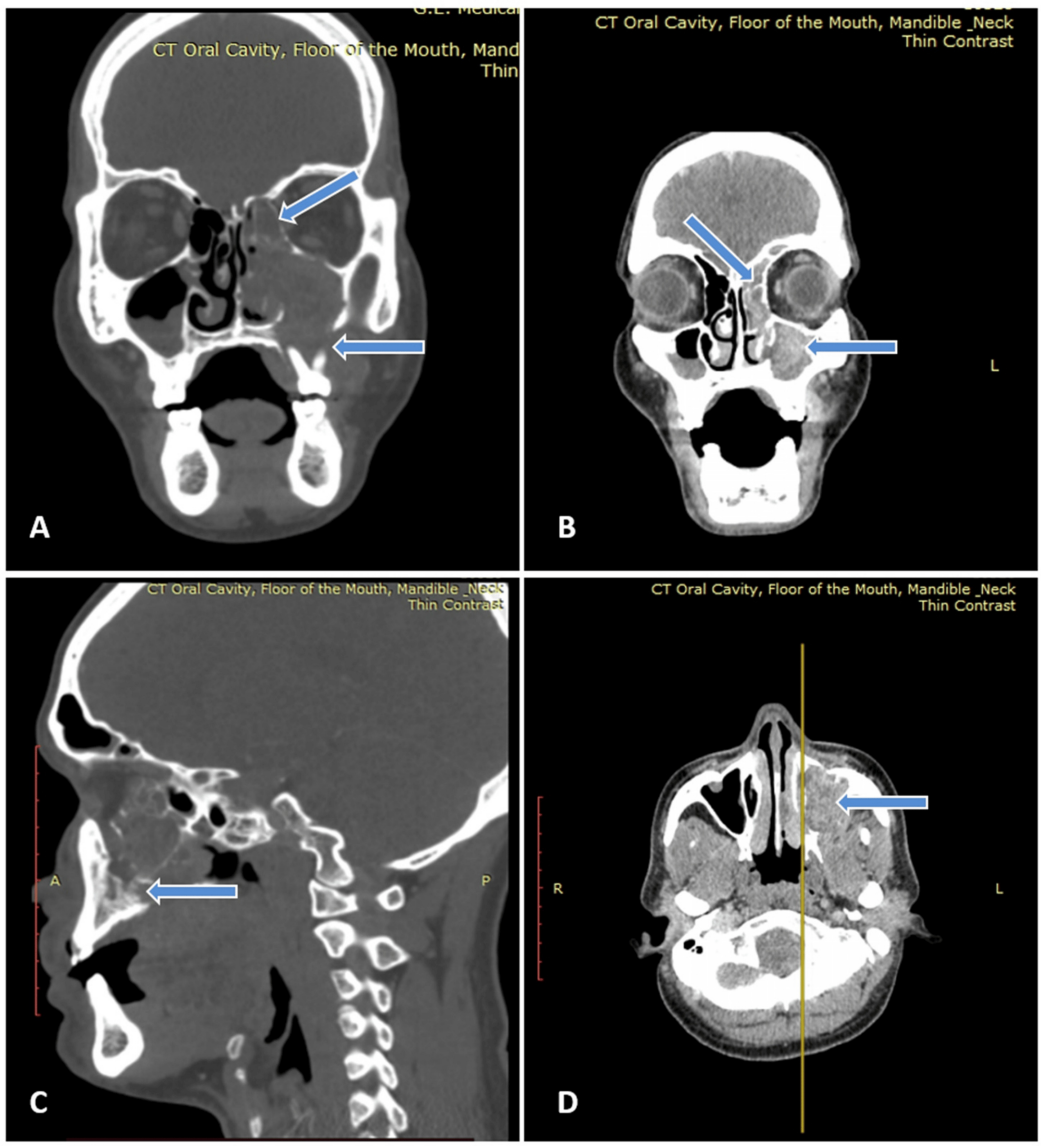
CECT show presence of hyper-attenuated area (arrow) in left side of maxilla involving antrum, ethmoid, and frontal sinus in coronal (A & B), sagittal (C), and transverse planes (D) CECT: contrast-enhanced computed tomography

Contrast-enhanced computed tomography (CECT) revealed the presence of a hyper-attenuated area in the left side of the maxilla involving the antrum, ethmoid, and frontal sinus (Figure 4). Bone destruction was noted in the maxilla at the medial, posterior, and posterolateral walls, involving the lateral pterygoid. Lymphadenosis was noted in level IB and II nodes, and level II lymph node swelling also showed metastatic features.

The patient was otherwise healthy, all routine hematological investigations were within normal limits, and chest X-rays revealed normal findings. He also tested negative for HIV I & II, hepatitis B surface antigen, and hepatitis C. Based on the clinical and radiological findings, a provisional diagnosis of SCC of the maxilla was made and was staged as T4b N1 M0 according to the clinical stage classification (cTNM) of the Eighth American Joint Committee on Cancer (AJCC) Staging System.

**Figure 5:**
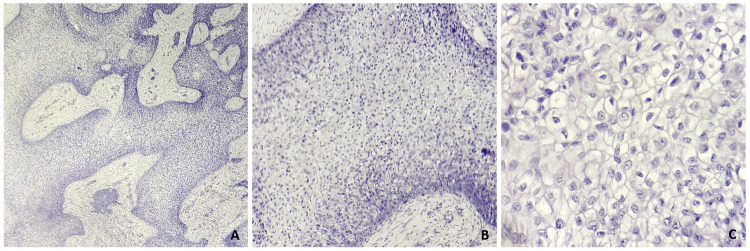
Photomicrographs show thick anastomosing strands of round to ovoid neoplastic cells with predominantly clear cytoplasm and marked cellular and nuclear pleomorphism infiltrating into the fibro-cellular connective tissue stroma viewed at 4x (A), 10x (B), and 40x magnification (C)

**Figure 6:**
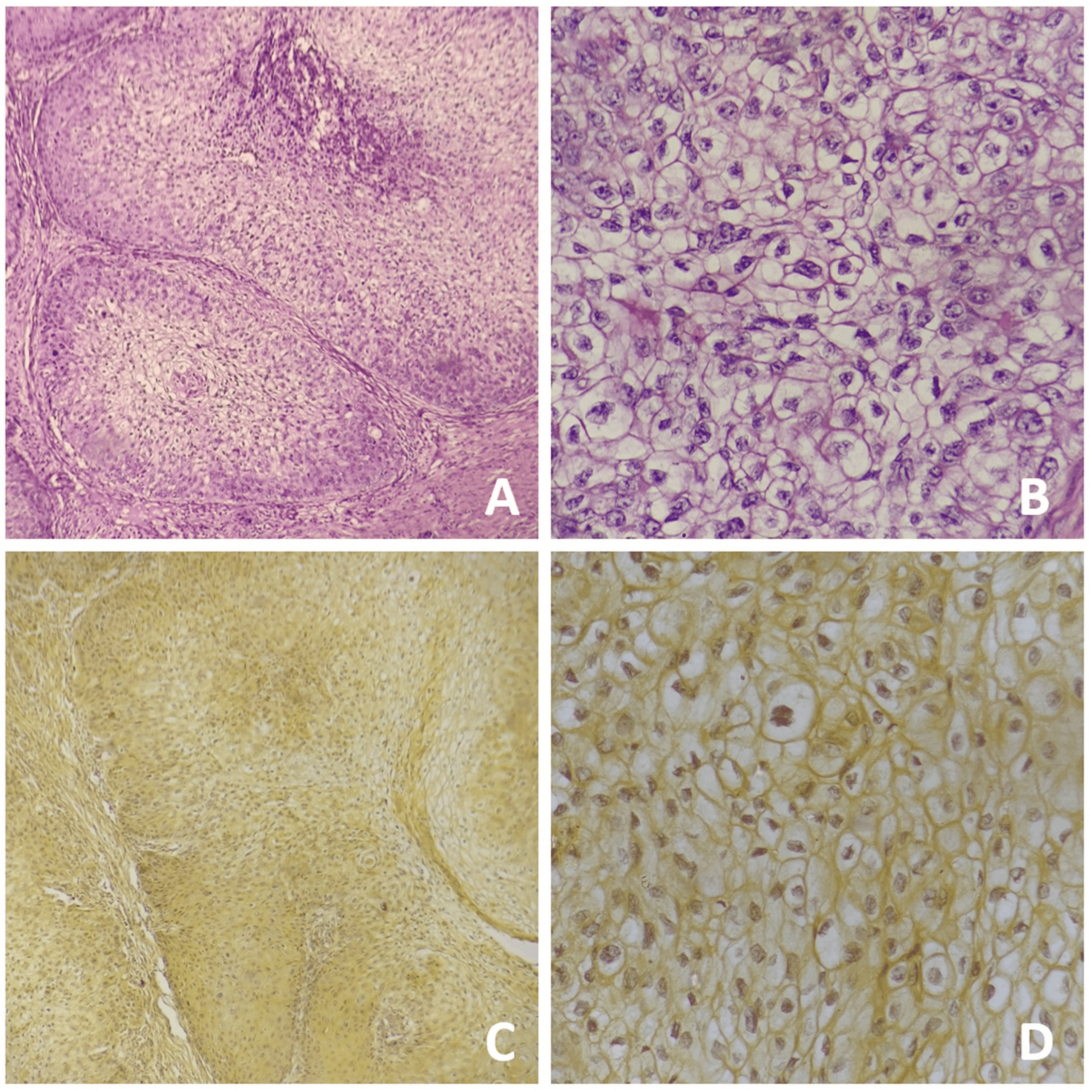
Photomicrographs show neoplastic cells yielding negative result for periodic acid Schiff (PAS) viewed at 4x (A) and 40x magnification (B), and mucicarmine viewed at 4x (C) and 40x magnification (D)

**Figure 7:**
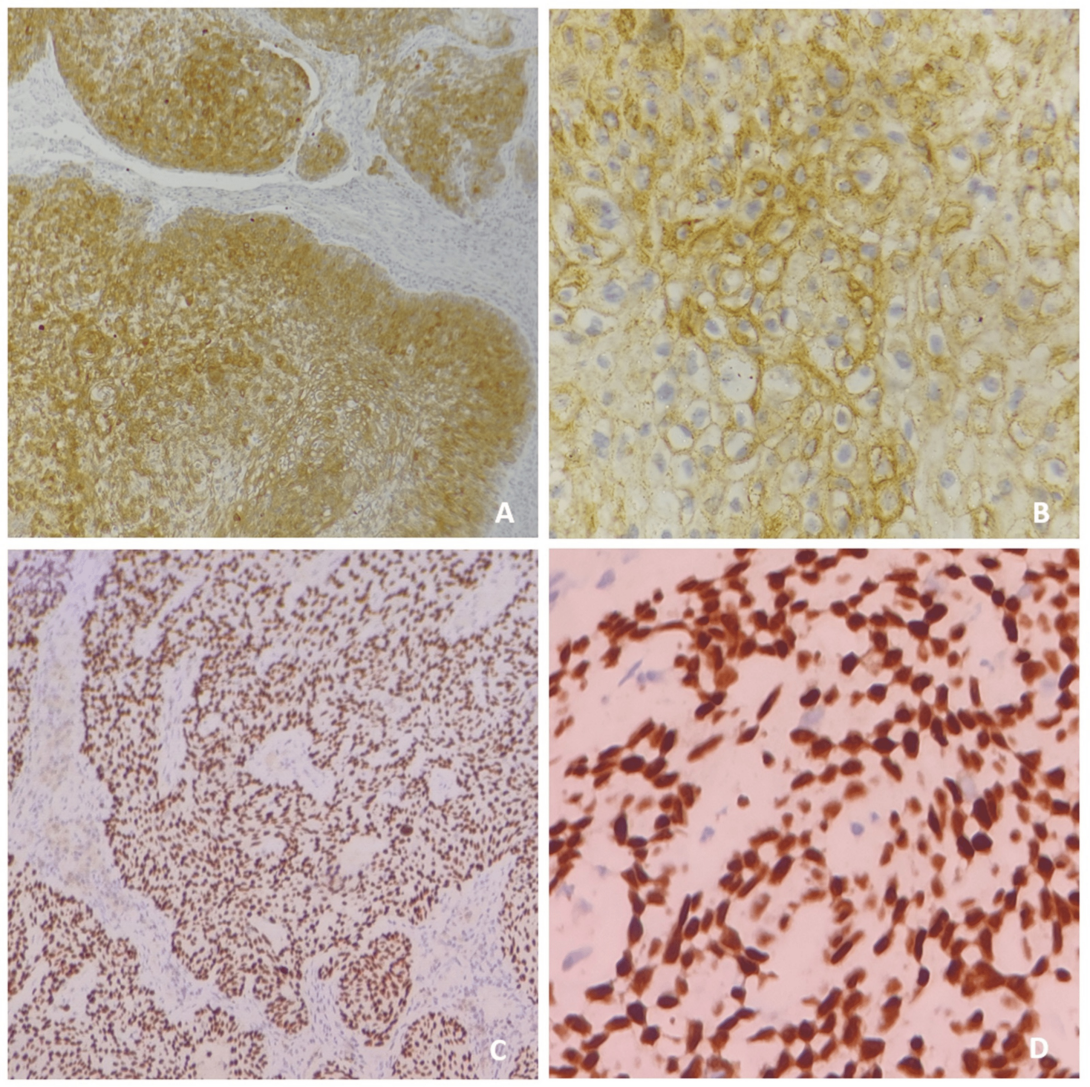
Photomicrographs show neoplastic cells that are strongly and diffusely immunopositive for pan-cytokeratin viewed at 4x (A) and 40x magnification (B), and P63 viewed at 4x (C) and 40x magnification (D)

Incisional biopsy was performed from the representative site of the lesion and histopathologically evaluated. H&E-stained sections revealed the presence of thick anastomosing strands of neoplastic cells infiltrating into the fibro-cellular connective tissue stroma. The neoplastic cells were round to ovoid in shape, with predominantly clear cytoplasm, and showed marked cellular and nuclear pleomorphism, nuclear hyperchromatism, altered nuclear-cytoplasmic ratio, and many cells showing vesicular nuclei (Figure 5). The histochemical analysis yielded negative results for periodic acid Schiff (PAS) and mucicarmine (Figure 6). Immunohistochemistry (IHC) studies revealed diffuse strong immunopositivity for pan-cytokeratin (Pan-CK) and P63 (Figure 7). Sections were negative for S100 and CD10. Based on clinical, radiographical, histopathological, histochemical, and IHC evaluation, a confirmatory diagnosis of a clear-cell variant of OSCC was made.

**Figure 8:**
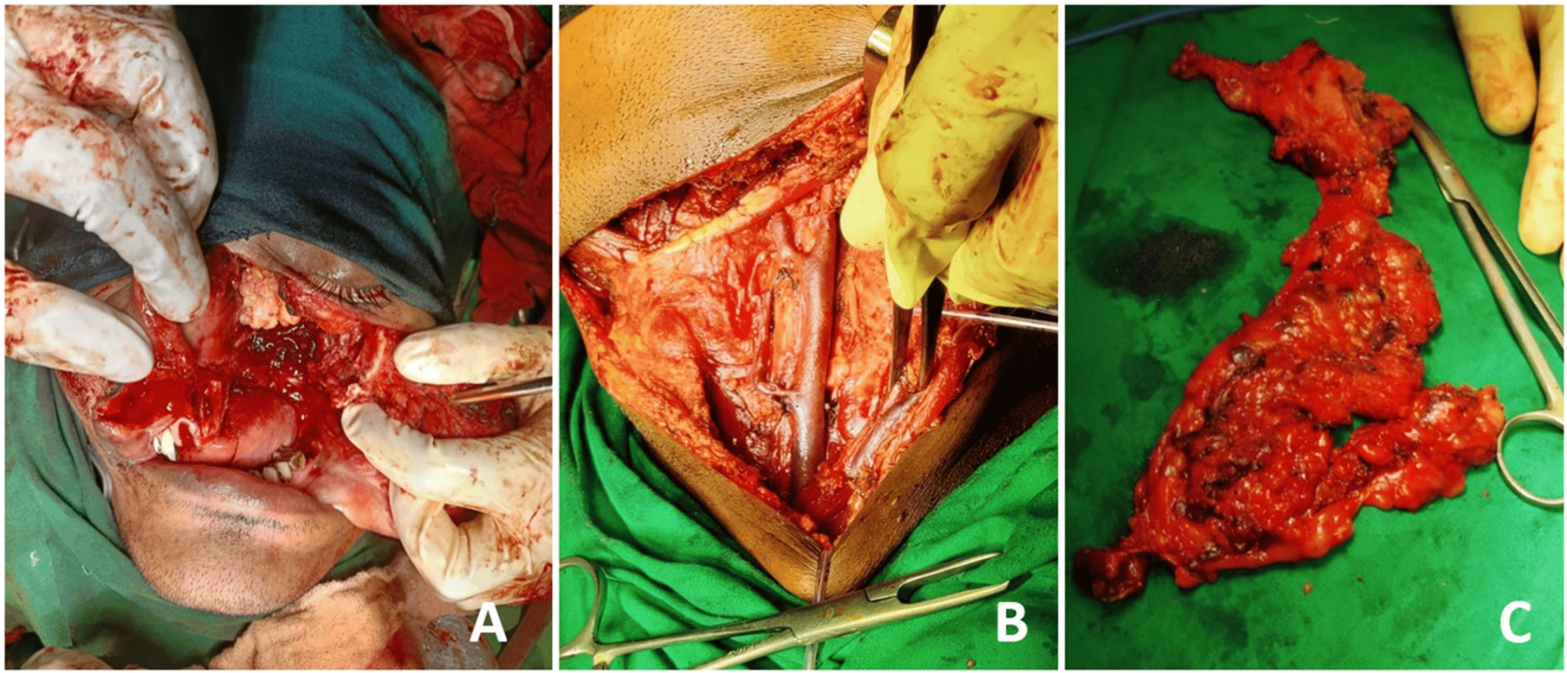
Intraoperative images of the patient undergoing wide local excision of lesion along with segmental maxillectomy with removal of associated sinus (A), ipsilateral modified radical neck dissection (B), and excised specimen of level I-V lymph nodes in fibro-fatty tissue (C)

**Figure 9:**
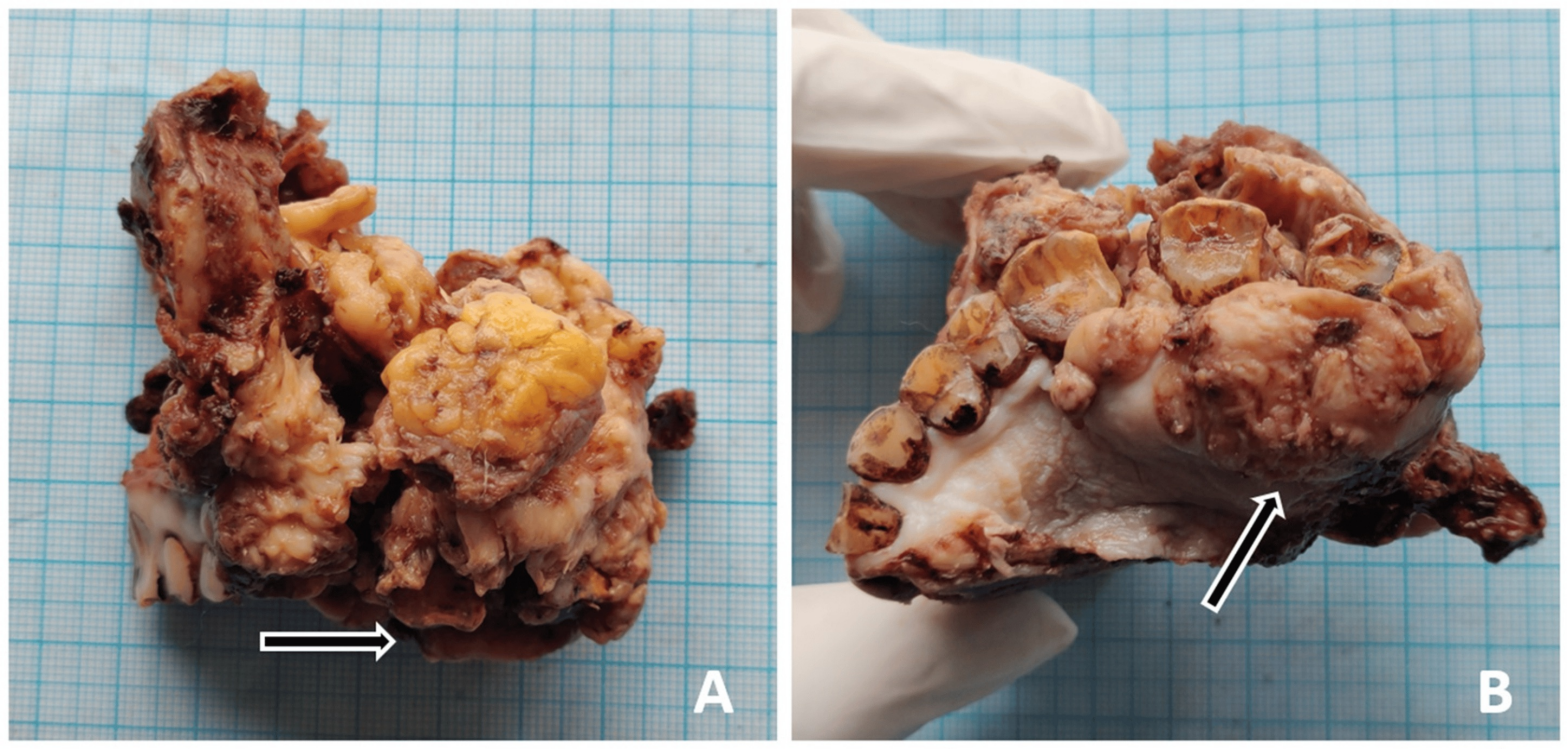
Gross composite specimen showing tumor proper and adjacent hard and soft tissue in side (A) and palatal (B) views

The patient was referred to the Department of Maxillofacial Surgery for further management. He underwent wide local excision of lesion along with segmental maxillectomy, removal of associated sinus, and ipsilateral modified radical neck dissection (Figure 8). The composite excised specimen (Figure 9) submitted for histopathological examination showed mucosal and bony margins to be free of tumor cells; however, the infiltrative pattern of invasion was noted in the bone underlying the tumor. The depth of invasion (DOI) was noted to be 9 mm. Sixty lymph nodes were removed from level I to level V in the specimen. The largest node measured 2.4 cm x 1.8 cm from level II and showed metastatic features without any extranodal extension, giving a lymph node ratio (LNR) of 1:60. Lymphovascular emboli, perineural invasion, giant cell response, sarcolemmal invasion were absent and worst pattern of invasion (WPOI) 3 was noted. The patient was advised to undergo adjuvant radiotherapy at a higher center. Unfortunately, we lost the patient for further follow-up.

## Discussion

Cells showing clear cytoplasm with a distinct nucleus in H&E-stained histologic sections are known as clear cells. These cells may be formed due to a variety of factors including artefactual changes, intracellular accumulation of fluids, glycogen, mucin, mucopolysaccharides, intermediate filaments, immature zymogen granules, phagocytized remnants of foreign bodies, hydropic degeneration, and scarce cellular organelles. Clear cell changes are also likely to develop in tumors due to clonal evolution [22].

Head and neck clear cell tumors constitute a diverse group of lesions having varied origins like odontogenic, salivary gland, metastatic, keratinocytic, melanocytic, osseous, cartilaginous, adipocytic, or adnexal [22]. Intraoral clear cell tumors can broadly be categorized as salivary gland neoplasms like mucoepidermoid carcinoma, acinic cell carcinoma, clear‑cell myoepithelial carcinoma, epithelial‑myoepithelial carcinoma, and hyalinizing clear‑cell carcinoma; odontogenic neoplasms like clear odontogenic ghost‑cell tumor, clear cell variant of calcifying epithelial odontogenic tumor, and clear‑cell odontogenic carcinoma, and metastatic tumors from renal cells, breast, lungs or liver [17, 21], among which lesions of salivary gland and odontogenic origin comprise about 90% of the cases [14]. All these lesions serve as probable differential diagnoses, thus presenting a diagnostic challenge to the pathologist, necessitating the use of histochemical and immunohistochemical evaluation.

Accumulation of intracellular glycogen and mucin are demonstrated by PAS and mucicarmine staining. Negative reactions with both stains significantly decreased the possibility of the present case being a salivary gland neoplasm [22]. Additionally, the lack of intermediate cells in the histologic sections eliminated mucoepidermoid carcinoma. Acinic cell carcinoma was ruled out due to the absence of PAS-positive, diastase-resistant secretory granules in the cytoplasm. The dearth of dense fibrous stroma excluded hyalinizing clear-cell carcinoma. S100 immuno-negativity precluded the probability of the lesion being acinic cell carcinoma, clear-cell myoepithelial carcinoma, and epithelial-myoepithelial carcinoma [6].

Furthermore, clear-cell odontogenic carcinoma was ruled out due absence of tumor islands displaying peripheral ameloblastomatous palisaded columnar cells or islands of hyperchromatic basaloid cells, and lack of PAS-positive glycogen-rich clear cells [21]. The clear-cell variant of calcifying epithelial odontogenic tumor characteristically demonstrates amyloid deposits and Liesigang ring calcifications, the absence of which negated its possibility for our case [11,23]. Clear odontogenic ghost-cell tumor was excluded as the sections did not show the presence of ghost cells with aberrant keratinization [15].

Clear-cell variant of renal cell carcinoma (RCC) accounts for 70-80% of all RCC cases and is composed of round to polygonal neoplastic cells having clear cytoplasm. These tumors are characterized by the presence of rich sinusoidal vasculature, prominent intra-tumoral hemorrhage, solid as well as cystic areas, and PAS-positive diastase-sensitive glycogen-containing clear cells [6,16]. The absence of these histologic features compounded with negative PAS reaction and immunonegativity for CD10 precluded the suspicion of metastatic RCC. Normal findings in the general examination, hematological evaluation, and chest X-ray ruled out the probability of metastasis from the liver or lungs to the oral cavity. Based on the above conclusions and positive IHC reaction for pan-CK and P63, we reached the final diagnosis of a clear-cell variant of OSCC.

In 1980, Kuo first reported a series of six cases to emphasize the existence of cutaneous clear-cell carcinoma as a distinct category of tumors, separate from sebaceous carcinoma or glycogenated squamous cell carcinoma. He believed these to be a variant of SCC undergoing hydropic degeneration and classified them according to their histologic patterns into three types, namely type I or keratinizing clear-cell carcinoma, type II or non- keratinizing clear-cell carcinoma, and type III or pleomorphic clear-cell carcinoma [4].

**Figure 10:**
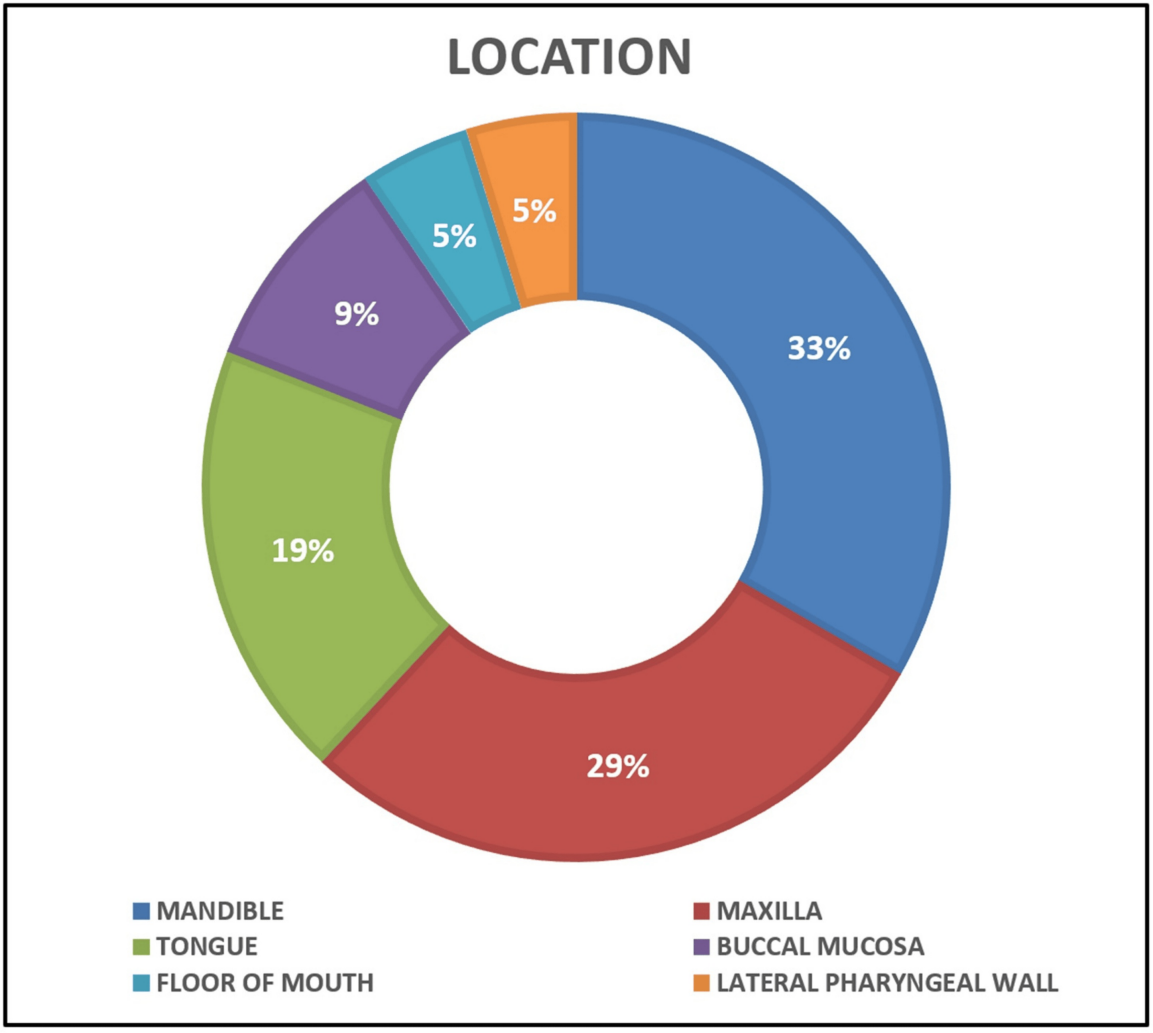
Pie diagram showing distribution of reported cases according to anatomical location of occurrence One synchronous case affected both maxilla and mandible Image Credits: Authors

Although OSCC is the most common malignancy of the oral cavity, an extensive search of the literature evinces the rarity of its clear-cell variant with only 19 reported cases (Table 1), among which one is synchronous [6]. Frazier et al., in 2012, presented the first case of a primary glycogen-rich clear-cell variant of SCC in the oral cavity arising from mandibular gingiva [5]. In the same year, Kumar et al. reported a case of glycogen-free, synchronous OSCC of the clear-cell variant [6]. Over the next 12 years, five cases showing the presence of glycogen and 12 cases without the presence of intracellular glycogen were noted. The present case shows a negative reaction for PAS and tumor cells are free of glycogen. Only five cases have been reported in the maxilla so far (Figure 10, Table 1), the current case being the sixth. The other locations reported are the mandible, tongue, buccal mucosa, floor of the mouth, and lateral pharyngeal wall with seven, four, two, one, and one cases, respectively (Table 1).

**Table 1:** Reported cases in the literature of clear cell variant of oral squamous cell carcinoma PAS: periodic acid Schiff; PAN-CK: pan-cytokeratin; EMA: epithelial membrane antigen; SMA: smooth muscle actin; HMB: human melanoma black

Author	Age/Sex	Location	Special Stains	Presence of Glycogen	IHC Analysis
Frazier et al., 2012 [5]	59/F	Posterior mandibular gingiva	PAS +ve PAS-D and Mucicarmine -ve	Present	-
Kumar et al., 2012 [6]	70/F	Anterior maxilla and posterior mandible (Synchronous)	PAS and Mucicarmine -ve	Absent	CK8, CK18, EMA +ve Vimentin, S-100, HMB-45 -ve
Nainani et al., 2014 [7]	52/M	Buccal mucosa	PAS and Mucicarmine -ve	Absent	CK8, CK18 +ve Vimentin, S-100 -ve
Romanach et al., 2014 [8]	60/F	Buccal mucosa extending to soft palate	PAS +ve	Present	CK AE1/AE3, p63 +ve CD 10 & Vimentin -ve
Kaliamoorthy et al., 2015 [9]	35/F	Postero-lateral border of tongue and lingual vestibule	PAS and Mucicarmine -ve	Absent	CK AE1/AE3 +ve SMA, HMB-45 -ve
Devi et al., 2017 [10]	55/M	Posterior maxillary ridge	PAS and Mucicarmine -ve	Absent	CK, EMA +ve
Khoury et al., 2017 [11]	66/F	Lateral tongue extending to floor mouth (anterior), oropharynx, and retromolar fossa (posterior)	PAS +ve PAS-D labile	Present	CK 5/6 +ve S-100, Calponin, SMA & HMB45 -ve
Kakoti et al., 2018 [12]	59/M	Upper jaw extending to upper lip and nasolabial fold	PAS -ve	Absent	CK +ve EMA, S-100 -ve
Sahni et al., 2019 [13]	40/F 45/M	47 region to retromolar area Lower jaw	Both cases were PAS and Mucicarmine -ve	Absent	-
Ramani et al., 2021 [14]	42/F	Mandibular alveolar mucosa	PAS and Mucicarmine -ve	Absent	CK +ve S-100, SMA, CD 117 -ve
Hasegawa et al., 2022 [15]	70/M	Ventral surface of tongue	PAS, Mucicarmine, and Alcian blue -ve	Absent	CK AE1/AE3 +ve CK5/6, CK17 weakly +ve
Sharma et al., 2022 [16]	55/M	Posterior maxilla bilaterally	PAS and Mucicarmine -ve	Absent	CD 10 -ve
Mukkanwar et al., 2022 [17]	60/M	Lateral border of tongue	PAS-D labile	Present	CK AE1/AE3 +ve
Apandi et al., 2022 [18]	65/M	Floor of mouth	PAS, PAS-D, Mucicarmine, and Alcian blue -ve	Absent	CK, p63 +ve CK 7, CK 20, S-100 -ve
Hirose et al., 2023 [19]	89/F	Maxillary alveolar ridge	PAS +ve PAS-D, Mucicarmine, and Alcian blue -ve	Present	CK AE1/AE3, CK5/6 & p63 +ve
Bharadwaj et al., 2023 [20]	71/M	Lateral pharyngeal wall	PAS-D labile	Present	PAN-CK +ve
Sharma et al., 2024 [21]	65/F 62/M	Mandibular alveolus Mandibular alveolar region	Both cases were PAS and Mucicarmine -ve	Absent	Both cases were CK, EMA +ve CD 10, HMB-45, S-100, Calponin -ve
Present case	32/M	Posterior maxillary gingiva and hard palate extending to buccal vestibule	PAS and Mucicarmine -ve	Absent	PAN-CK, p63 +ve S100, CD10 -ve

**Figure 11:**
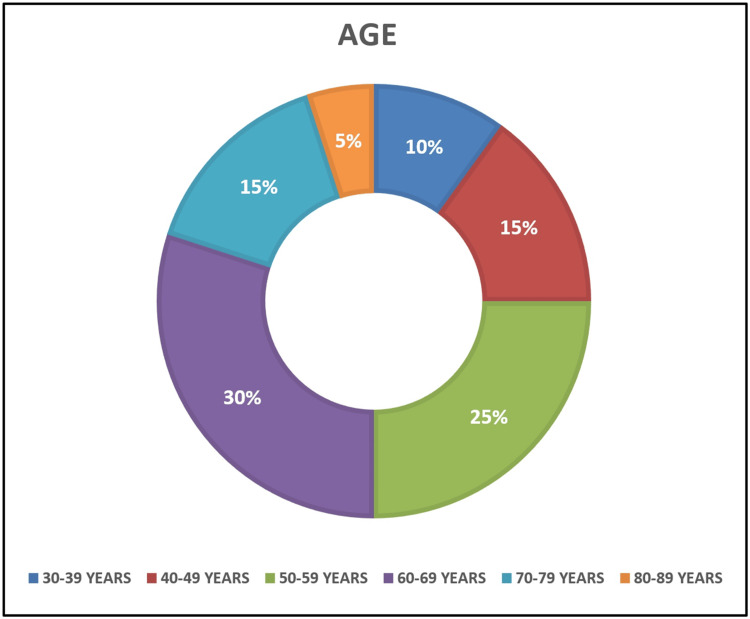
Pie diagram showing distribution of reported cases according to age of occurrence Image Credits: Authors

The majority of the cases have been reported in older individuals (Table 1) with the highest prevalence in the seventh decade (Figure 11), while the present case has been reported in a young patient. It is also the youngest recorded case of a clear-cell variant of OSCC to date, thus reinforcing its uniqueness. Although previous reports state a marked female preponderance [12,17], a shift has been noted showing an almost equal sex predilection with male:female ratio being 1.2:1. Majority of the previously reported cases are associated with poor prognosis and authors opine that the clear-cell variant of OSCC is extremely aggressive in nature. In accordance with such findings, the present case too demonstrated an aggressive clinical course and lymph node metastasis, thus indicating an unfavorable outcome.

## Conclusions

Clear-cell variant of OSCC is an astoundingly uncommon neoplasm with a wide array of histologic mimickers, thus making its diagnosis challenging and elusive. It is essential to characterize and distinguish this rare variant from other clear cell lesions having different origins. Further reporting and research can assist in better comprehension of the etiology, pathogenesis, clinical behavior, and outcome of this rare entity. Clear-cell variant of OSCC is generally associated with an aggressive clinical course and dismal prognosis. Meticulous assessment of clinical, histopathological, histochemical, and immunohistochemical findings is therefore a sine qua non for early diagnosis and timely treatment of this killer disease. 
